# Presence of keel bone damage in laying hens, pullets and roosters of local chicken breeds

**DOI:** 10.1371/journal.pone.0297586

**Published:** 2024-01-26

**Authors:** Lisa Jung, Sonja Hillemacher, Inga Tiemann, Mascha Lepke, Dirk Hinrichs

**Affiliations:** 1 Animal Breeding Section, University of Kassel, Witzenhausen, Germany; 2 Institute of Agricultural Engineering, University of Bonn, Bonn, Germany; University of Life Sciences in Lublin, POLAND

## Abstract

In commercial laying hens, keel bone damage (KBD) is a severe health and welfare problem leading to pain, reduced mobility and decreased laying performance. Flocks of all production systems and hybrid lines can be affected. KBD is a multifactorial welfare issue and, among other factors, associated with a high laying performance which negatively affects the calcium deposit in the medullary bones. Therefore, mature hens of local breeds with much lower egg production than commercial hybrids may be expected to show less or even no keel bone damage. This study evaluates (i) the prevalence of KBD in local breeds, (ii) the difference in type and level of damages, and (iii) if roosters and pullets are also affected. In total, we palpated 343 mature hens, 40 pullets, and 18 roosters of 13 different local breeds and one commercial hybrid. The animals were kept on eight different farms in free-range or floor-housing systems. Our results showed that on average 44.2% of mature hens per local breed were affected by KBD (range: 11.1%-84.7%). We found deviation of less than 1 cm in 26.9%, deviations of more than 1 cm in 6.4% and palpable fractures in 23.8% of the mature hens of local breeds. The tip was damaged in 23.6% of the mature hens. Also, pullets and roosters were affected by KBD. Finally, we found that KBD also occurs in local breeds. Therefore, we conclude that even the low laying performance of local breeds does not prevent them from the occurrence of KBD.KBD in local breeds may rather be associated with genetics (breed) as well as management and housing. Thus, breeders of local breeds should include bone health as a selection trait. Owners of local breeds should also pay attention to the condition of the keel and ought to be trained about preventive measures.

## Introduction

One of the most severe welfare problems in poultry production is keel bone damage (KBD) predominantly in laying hens [1]. KBD is found in every flock, with a prevalence of 3% to 99% affected hens, regardless of the commercial line in use or the housing system applied [2–5]. Even in laying hens kept for organic production in free-range, the prevalence is high [6], although free-range use is generally assumed to support bone stability by providing space for movement on the one hand and daylight which improves vitamin D synthesis on the other hand [7].

KBD is influenced multifactorial, and the underlining principles are not fully understood yet. In addition to the laying performance, multiple causes have been found, like lack of exercise [8], but also genetics [9–12], feeding [12], and the influence of housing and rearing systems [12–15]. KBD can be divided into fractures and deformations. The deformation of the keel bone describes any deviation from the straight axis [16], whereas fractures can be associated with dislocations or callus deposits [17]. Fractures may also occur specifically at the keel bone tip, defined as the caudal last 1 to 2 cm [14, 18, 19]. Tip fractures should be distinguished from medial fractures [8] because the tip is the most fragile part [14], at which ossification is completed latest between week of age 30 to 40 [20]. Therefore, it is assumed that the early onset of lay before ossification can negatively affect the prevalence of tip damage [8]. Also early egg size might be a factor driving this specific alteration [21, 22]. In general, a high laying performance of commercial lines is expected to be one of the main influencing factors [4, 5, 23].

Due to domestication, the laying performance was increased by more than tenfold, from two clutches of 12 eggs per year in the red junglefowl (*Gallus gallus*) to more than 300 eggs per year for a commercial layer [24]. This egg production on a continuously high level throughout the laying period requires calcium for eggshell formation which is typically provided in the feed. In case of overuse, the hen mobilizes calcium from medullary bones by osteoclastic resorption which in turn weakens bone stability [25, 26]. Dunn et al. [27] concluded from their studies focusing on the relationship of egg production, bone quality, and genetics that sexual maturity is a key factor leading to the assumption that an adequate start of lay may offer an opportunity to improve overall bone health in layers. A further conclusion is that improving bone quality should not necessarily result in lower egg production or egg quality. Nevertheless, numerous of the proposed causal mechanisms underlying KBD are linked to a selection for efficient production. In their investigation on four purebred breeds kept in cage systems, Kittelsen et al. [28] found four fractures in 126 keel bones, three of them in the most inventively selected breed with the earliest onset of lay. Likewise, in a pilot study in red junglefowl (*Gallus gallus*), Kittelsen et al. [29] found no fractures in 17 roosters and 1 fracture in 12 hens, and 1 rooster and 10 hens with very slight deviation.

In the context of welfare, preventing health problems and pain is one main target, and supporting natural behavior another. Several authors found changes in natural behaviors in chicken affected by KBD, like decreased mobility [30] and increased preening [31], as well as indications of pain when hens were affected by keel bone fractures [1, 32, 33].

To support welfare also beside commercial poultry production, this study aims to evaluate (i) the prevalence of KBD in 13 local and 1 commercial chicken breeds, (ii) the difference in kind and level of KBD and (iii) if KBD also occurs in pullets and roosters.

Knowledge on the prevalence of KBD in local breeds should initiate the awareness of welfare-associated problems also in lower performing breeds.

## Animals, materials and methods

### Institutional review board statement

The study was carried out in accordance with the German animal protection act (implementing the Directive 2010/63/EU on the protection of animals used for scientific purposes) and the Guidelines for Ethical Treatment of Animals in Applied Animal Behavior and Welfare Research of the International Society for Applied Ethology (2017). The hens were reared and kept for production meant for human consumption according to national law and guidelines, and not for experimental purposes. They did not undergo any experimental procedure and were only monitored by non-invasive clinical scoring.

**Fig 1:**
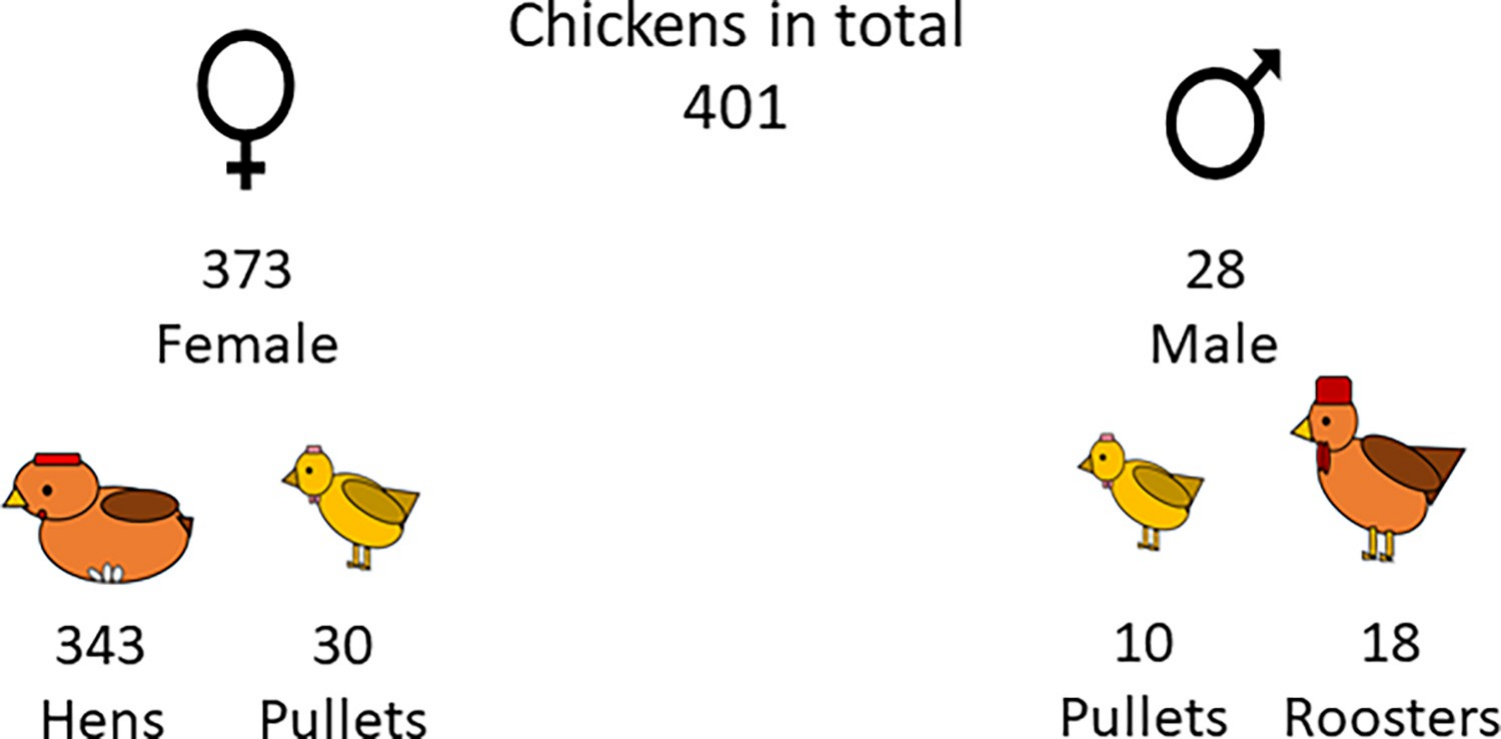
Number of in the investigation included mature hens, roosters and pullets.

In total, we included 401 animals from 13 different local breeds and 17 animals from one commercial hybrid that were kept on eight farms in the investigation. Fig 1 shows the number of included hens, roosters and pullets, whereby roosters and hens with less than 18 weeks of age are counted as pullets in the analyses.

Included breeds were: Augsburger, Australorps, Brabançonne, German Sperber, Lakenfelder, Lohmann Brown, Marans, Malines, East Frisian Gull, Vorwerk, Bielefelder, Dresdner, Sulmtaler and Sundheimer bantam. Table 1 shows the number of animals per farm and breed.

**Table 1:** Number of animals per farm and breed.

Farm Breed	F1	F2	F3	F4	F5	F6	F7	F8	Total n/breed
Augsburger						9			9
Australorps						7			7
Bielefelder					5	1		54	60
Brabançonne							9		9
German Sperber				64					64
Dresdner					7	7			14
Lakenfelder			15						15
Lohmann Brown					5	12			17
Marans					18	19	20		57
Malines	10						33		43
East Frisian Gull					9	66			75
Sulmtaler	3						11		14
Vorwerk		3	9						12
Sundheimer bantam			5						5
Total n/farm	13	3	29	64	44	121	73	54	401

In Lakenfelder, we only examined pullets (Table 2).

**Table 2:** Number of investigated animals, distinguished in mature hens, pullets and roosters per breed.

Breed	n	Mature hens	Pullets^1^	Roosters
Augsburger	9	8		1
Australorps	7	7		
Bielefelder	60	59		1
Brabançonne	9	9		
German Sperber	64	63		1
Dresdner	14	14		
Lakenfelder	15		15	
Lohmann Brown	17	16		1
Marans	57	43	13	1
Malines	43	41		2
East Frisian Gull	75	66		9
Sulmtaler	14	13		1
Vorwerk	12		12	
Sundheimer bantam	5	4		1
Total	401	343	40	18

^1^ Hens that were not yet laying eggs

Performance data of the breeds, as reported in the literature, are presented in Table 3. Seven of the breeds were white layers, six were brown layers and one was a commercial brown layer. The laying performance of the breeds varies between 140 and 220 eggs/year according to the literature, taking the commercial hybrid (321 eggs/year) not into account (Table 3).

**Table 3:** Laying performance, egg weight, eggshell color and start of lay for 12 local chicken breeds.

Breed^1^	Eggs/year^2^	Egg weight^2^	Eggshell colour
	[n]	[g]	[brown/white]
Augsburger	180	60	white
Australorps	200	55	brown
Bielefelder	220	60	brown
Brabançonne	180	60	white^1^
German Sperber	140	60	white
Dresdner	180	55	brown
Lakenfelder	200	55	white
Lohmann Brown	321	63	brown
Marans	180	60	brown
Maline	160	60	brown
East Frisian Gull	160	60	white
Sulmtaler	180	55	brown
Vorwerk	180	60	white
Sundheimer bantam	160	45	brown

^1^[34], ^2^[35]

The majority of animals had access to free-range, except for the 54 Bielefelder of farm F8, which were housed in a floor-housing system with access to a covered veranda. Animals of mobile housing systems with free-range were categorized as free-range housing system. All animals had access to wooden perches, perch heights differed between farms. Parameters recorded for each animal were breed, sex, age (pullet or adult), farm, group size, housing system, perch height, body weight, and keel bone condition (see definitions below). Although week of age plays an important role in the prevalence of KBD, this information was not available in the current study. Therefore, we only differentiated between pullets (less than 18 weeks of age), mature hens and roosters. The group size of the different flocks ranged between 3 and 200 individuals with an average of 104 animals (median: 70).

Keel bone condition was assessed once for each animal according to the scoring scheme shown in Table 4. A palpable fracture was assessed as present or not, while a deformation was additionally differentiated between two degrees of severity. During palpation, the main keel bone and the tip (last 2 cm) were assessed by running two fingers down the edge of the keel bone to detect alterations like deviations, palpable fractures, dislocations or proliferations.

**Table 4:** Scoring scheme for the palpation of KBD in laying hens, differentiated for deformations and fractures.

Indicator	Definition
Deformation	
Score 0	Straight, no deformation
Score 1	Deviation of straight axis ≤ 1 cm
Score 2	Deviation of straight axis >1 cm
Palpable fracture	
Score 0	No callus/pieces of fractured bone or dislocations palpable
Score 1	Callus/pieces of bone palpable
Tip (last 2 cm caudal)	
Score 0	No callus/pieces of fractured bone palpable, no compression or angle
Score 1	Callus/pieces of fractured bone palpable, compressed or angled

Weight was documented using a scale (Kern ECE 10K 5, d = 5g, Munich, Germany).

Statistical analysis was performed using the software R (version 4.2.2, R Core Team, 2020). Due to the low sample sizes, data of rooster and pullets was included only in descriptive analysis as well as data of farm F8 as this was the only farm with a floor housing system without free-range access.

We investigated the effect of the recorded variables on KBD on a subset of the data (mature hens with free-range access) by performing a generalized linear mixed-effect model (GLMMs) assuming a binomial distribution (Likelihood-ratio tests (LRT) with chi-squared tests were applied). The packages “lmerTest” and “emmeans” were used. Global p-values were calculated for each effect which was included in the model.

Prior to running the GLMM, correlations between variables were calculated using eta-coefficient for nominal/metric variables and Cramer’s V for nominal variables. In case of significant correlations (p ≤ 0.05) or strong correlations with an eta-coefficient above 0.8, one of the two variables was excluded as explanatory variable from the model. This resulted in including the variable farm as random effect in the model and the variables breed and perch height (breed vs. perch height, Eta = 0.363) as fixed effects (explanatory variables). The variables group size (Eta = 0.837), housing system (V = 0.807, p<0.001) and body weight (Eta = 0.815) were excluded from the model due to their strong correlations with the variable breed. The correlation between sex and breed was V = 0.345, p<0.001 and between age and breed V = 0.849, p<0.001. For comparing the prevalence of KBD between brown and white layers a chi-squared test was applied.

## Results

**Fig 2:**
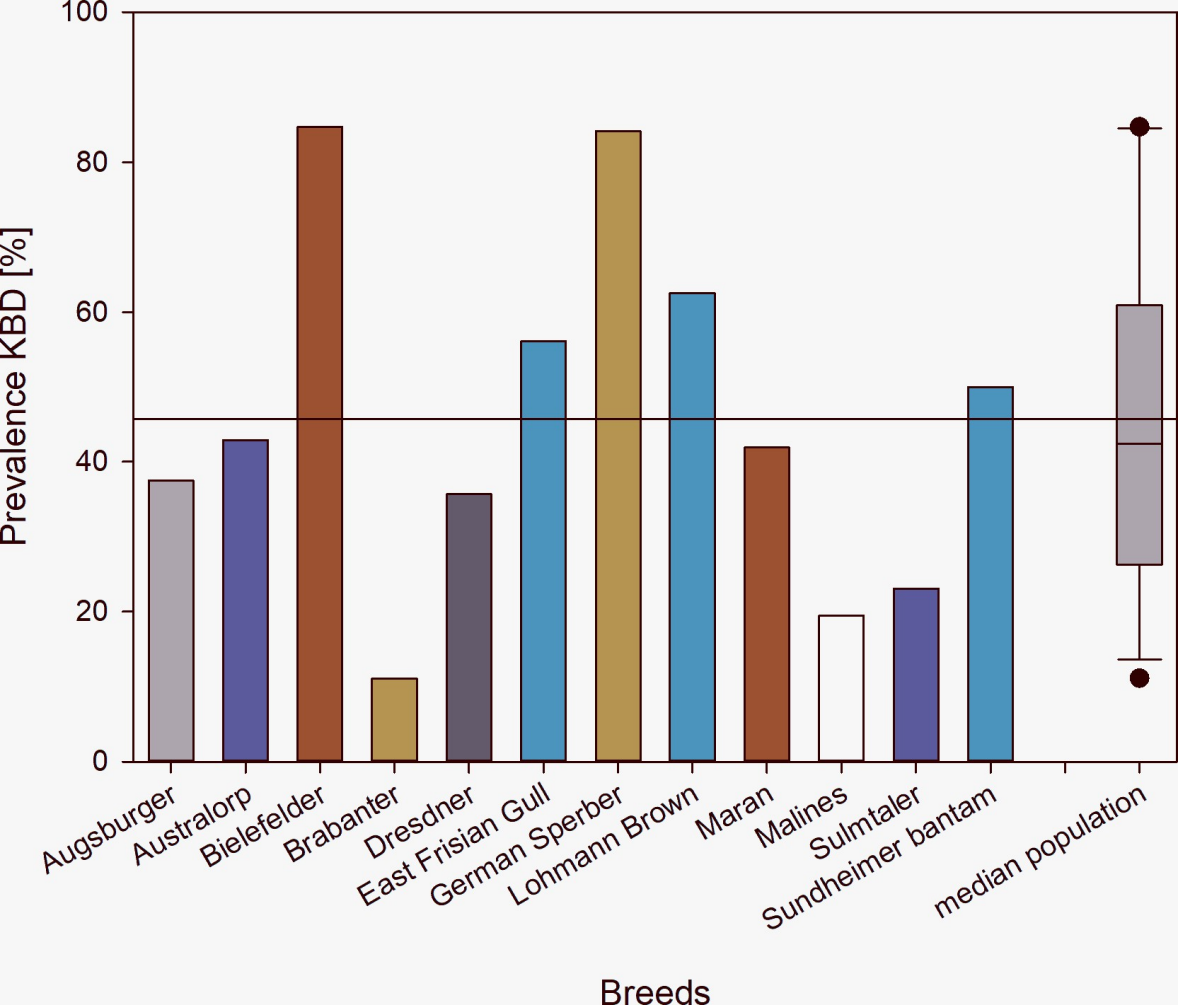
Keel bone damage (KBD) prevalence in % given for mature hens of 11 local breeds and 1 commercial hybrid (Lohmann Brown) in alphabetical order. Shown is also the overall mean ± standard error, also indicated as reference line.

This study showed that KBD also occurs in mature hens, pullets and roosters of local breeds with lower laying performances than commercial hybrids. Of all mature hens of the local breeds investigated, 56.0% of the hens were affected by KBD, whereas 44.2% hens, on average, per local breed showed KBDs (range: 11.1%-84.7%; Fig 2). 62.5% of the hens of the commercial hybrid were affected by KBD.

More detailed, 28.7% of the mature hens of local breeds showed score 1 deformations of the keel bone, 12.2% score 2 deformations, and 33.0% showed palpable fractures, compared to 25.0% score 1 deformations, 0% score 2 deformations and 43.8% palpable fractures in Lohmann Brown hens. The tip was damaged in 34.2% of the investigated hens of local breeds compared to 50.0% of the Lohmann Brown hens. Of the mature hens of local breeds, 21.4% were affected by all three kinds of keel bone damages: deformations, palpable fractures and damages of the tip (for comparison: 12.5% of Lohmann Brown hens).

The mature hens of the breeds Bielefelder and German Sperber showed the highest occurrence of KBD with 84.7% and 84.1%, respectively (Table 5).

**Table 5:** Prevalence (%) of KBD, score 1 deformations, score 2 deformations, fractures and damages of the tip for the different breeds separated by mature hens, pullets and roosters.

	Breed	n	KBD [%]	Deformations score 1 [%]	Deformations score 2 [%]	Fracture [%]	Tip [%]
*Mature hens*	Augsburger	8	37.5	25.0	0.0	12.5	0.0
	Australorps	7	42.9	28.6	0.0	14.3	14.3
	Bielefelder	59	84.7	39.0	33.9	66.1	64.4
	Brabançonne	9	11.1	0.0	0.0	0.0	11.1
	Dresdner	14	35.7	33.3	0.0	35.7	14.3
	East Frisian Gull	66	56.1	43.9	17.1	34.8	33.3
	German Sperber	63	84.1	42.4	19.7	44.4	60.3
	Lohmann Brown	16	62.5	28.6	0.0	43.8	50.0
	Maran	43	41.9	68.8	0.0	16.3	11.6
	Malines	41	19.5	11.6	0.0	4.9	2.4
	Sulmtaler	13	23.1	0.0	0.0	7.7	23.1
	Sundheimer bantam	4	50.0	3.2	0.0	25.0	25.0
*Pullets*	Lakenfelder	15	20.0	13.3	0.0	6.7	0.0
	Maran	13	7.7	7.7	0.0	0.0	0.0
	Vorwerk	12	41.7	8.3	0.0	33.3	0.0
*Roosters*	Augsburger	1	0.0	0.0	0.0	0.0	0.0
	Bielefelder	1	0.0	0.0	0.0	0.0	0.0
	East Frisian Gull	9	33.3	22.2	0.0	11.1	0.0
	German Sperber	1	100.0	100.0	0.0	100.0	100.0
	Lohmann Brown	1	100.0	100.0	0.0	0.0	0.0
	Maran	1	100.0	0.0	0.0	100.0	0.0
	Malines	2	0.0	0.0	0.0	0.0	0.0
	Sulmtaler	1	0.0	0.0	0.0	0.0	0.0
	Sundheimer bantam	1	100.0	0.0	0.0	100.0	0.0

**Fig 3:**
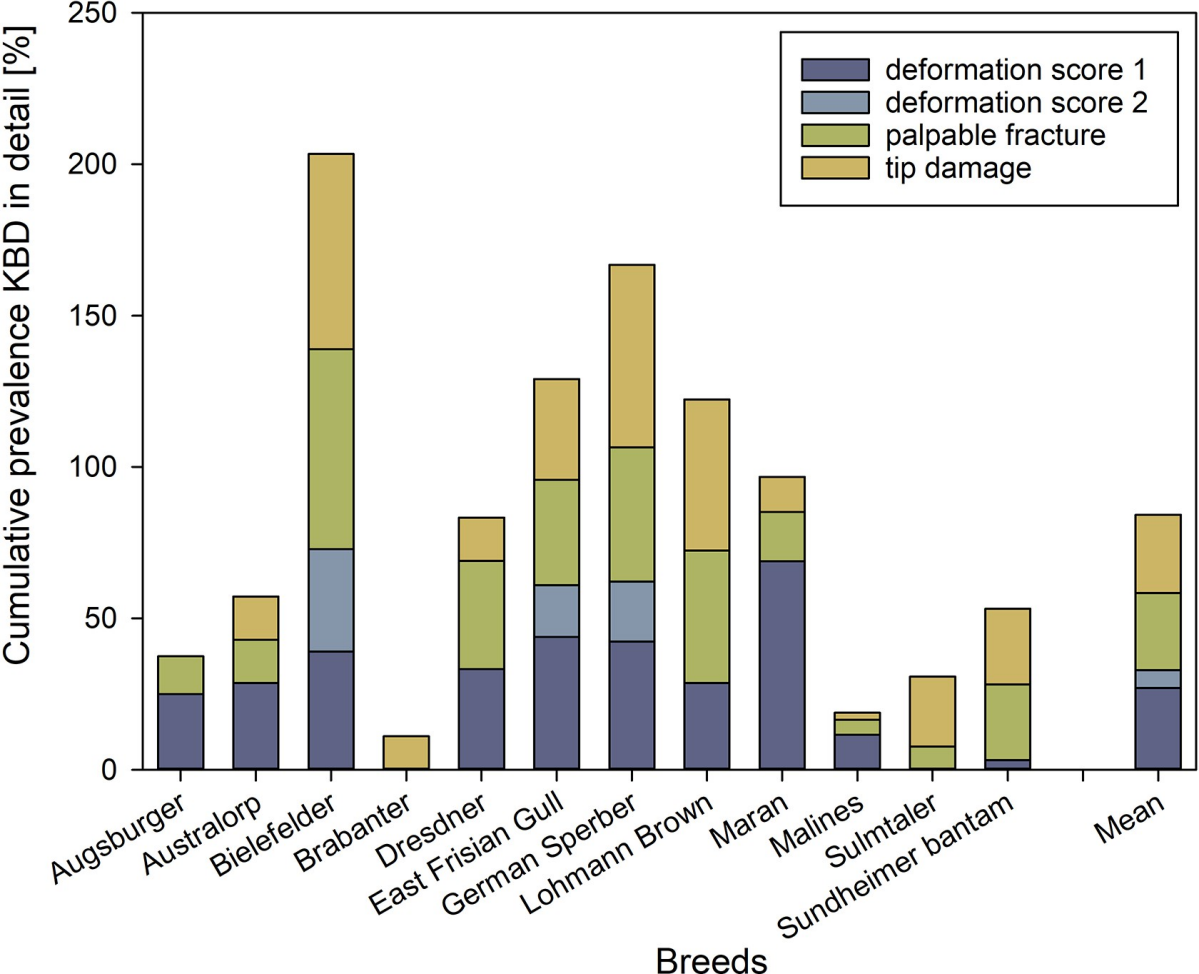
KBD in detail given in % per breed. Shown are deformations of score 1 and 2, palpable fractures, and tip damage of mature hens. Stacked bars do not sum up to 100% as the same bird might show several damages and will, in case, be counted several times.

Bielefelder hens also showed the highest amount of score 2 deformations (33.9%), followed by German Sperber hens with 20.6% and East Frisian Gull hens with 10.6% severe deformations of the keel bone (Fig 3). The other breeds were not affected by score 2 deformations. The highest prevalence of palpable fractures in mature hens was found for the breed Bielefelder with 66.1% of the animals being affected, again followed by mature hens of German Sperber (44.4%) and East Frisian Gull (34.8%). We found the same pattern of damage of the tip with 64.4% in Bielefelder hens, 60.3% in German Sperber hens and 33.3% in East Frisian Gull hens.

For the prevalence of KBD in pullets, data of three breeds, Marans (n = 13), Vorwerk (n = 12) and Lakenfelder (n = 15), were analysed (Table 5). We found a lower prevalence in pullets than the mature hens; on average, 22.5% of the pullets were affected by KBD. In 10% of the pullets, deviations of score 1 were found, and 12.5% showed palpable fractures. These fractures were only found in Lakenfelder and Vorwerk pullets, not in Maran pullets. No pullet showed a tip damage or deviations of score 2.

The average prevalence of KBD in roosters was 38.9%, whereas four breeds did not show any KBD maybe due to low sample size (Augsburger n = 1, Bielefelder n = 1, Malines n = 2, Sulmtaler n = 1, Table 5). Two roosters of the East Frisian Gull (n = 9), one German Sperber (n = 1) and one Lohmann Brown rooster (n = 1) showed score 1 deformations of the keel bone. Deformations of score 2 did not occur in any of the 18 investigated roosters. One rooster each of East Frisian Gull (n = 9), German Sperber (n = 1), Marans (n = 1), and Sundheimer bantam (n = 1) was affected by palpable fractures. The average prevalence of palpable fractures in roosters was 22.2%. One rooster (German Sperber) showed a palpable fracture and a deformation, and also a damaged. We did not find any severe deviations of score 2 in roosters.

For the subset of mature hens (including Lohmann Brown hens) in free-range housing systems (n = 289), we found the explanatory variable breed affecting significantly the prevalence of KBD (LRT, χ^2^(11) = 20.659, p = 0.037, see https://doi.org/10.48662/daks-31, Table S1 and R-script). German Sperber showed a significantly higher prevalence of KBD than Brabançonne (z = 3.768, p = 0.009), Dresdner (z = 3.417, p = 0.031), Maran (z = 4.795, p<0.001), Malines (z = 6.220, p<0.001), East Frisian Gull (z = 3.962, p = 0.004) and Sulmtaler (z = 4.300, p = 0.001). Also, East Frisian Gull hens had a significantly higher prevalence of KBD than Malines (z = 3.455, p = 0.027). Furthermore, perch height affected significantly the prevalence of KBD in mature hens in free-range housing systems (LRT, χ^2^(1) = 4.047, p = 0.044, see https://doi.org/10.48662/daks-31, Table S1 and R-script) with a higher prevalence of KBD with increasing perch height (estimate = 0.014).

When comparing the prevalence of KBD between brown and white-laying mature hens of local breeds, we found significant differences (χ^2^(1) = 5.795, p = 0.016) with white layers (mean KBD-score: 0.59, median: 1.0) having a higher prevalence for KBD than brown layers (mean KBD-score: 0.46, median: 0.0).

## Discussion

In the present study, we assessed the keel bone status of 343 mature hens, 40 pullets, and 18 roosters of 13 local and 1 commercial breeds by palpation. Overall, more than half of the investigated mature hens showed KBD. The prevalence of KBD in mature hens was more than two-fold for pullets as for rooster.

Although palpation is a common technique to assess KBD, the accuracy is very limited, despite the experience of the assessor in palpation. In our study, palpation was conducted by two trained persons that frequently carry out intra- and inter-observer reliability tests in commercial layer lines with acceptable to very good results. However, the inter-observer reliability is mostly poor for the tip, and palpation is not as sensitive and specific as x-ray or the evaluation of dissected bones [19]. Additionally, we assume that palpation is more difficult in local and dual-purpose breeds due to the thicker fat layer and the different anatomy.

In total we found a prevalence of KBD in the mature hens of local breeds with on average 44.2% in the range of KBD proportions in hens of commercial laying strains found in literature with between 3% to 99% affected hens [2–5], but lower prevalence than in the Lohmann brown flock included in our study with 62.5% affected hens. Nonetheless there was a high breed specificity.

The prevalence of palpable fractures in mature hens of different breeds varied from 0% to 66.1%, with the highest prevalence found in the breed Bielefelder, which was the only breed that was kept in a floor-housing system with no free-range access (54 of 59 Bielefelder hens were affected). In this specific case it remains unclear whether there is a breed predisposition or a risk factor associated with floor housing as both factors are highly confounded. Independent of the Bielefelder, in most breeds of our study the prevalence of palpable fractures was lower than in other studies which report the finding of fractures in approximately 52.2% of hens kept in floor systems [36]. However, the number of animals per breed and flock varied highly in our study (Table 1) and may have biased the results. Especially in the case of fractures we do not know if some of the breeds are prone to have minor fractures such as spontaneous ones, that are not palpable.

Rufener and Makagon [36] showed a higher prevalence of fractures in brown layers compared to white layers. In this investigation, eight brown layers were included: Sundheimer Bantam, Bielefelder, Sulmtaler, Marans, Australorps, Dresdner, Augsburger and Lohmann brown. In all eight brown-laying breeds, KBD was found in similar amounts to the white layers.

The prevalence of tip damage in mature hens (23.6%) was lower compared to those found in other studies on commercial flocks with e.g., 44.3% in Heerkens et al. [3], or 90.2% in Tracy et al. [19]. These differences may be due to low sample sizes in some of the breeds in our study which is a serious problem when working on breeds covered by the red list of endangered livestock and poultry species [37].

The prevalence of deformation in mature hens was comparable to commercial lines. We found score 1 and 2 deformations in almost every second hen. The highest prevalence of deformations was found in Bielefelder for which also the highest laying performance with around 220 eggs/year is reported in literature. The second highest prevalence of deformations of score 1 and 2 was observed for German Sperber with a laying performance of 180 eggs per year, followed by Sundheimer Bantam with a laying performance of 160 eggs. Malines showed comparable low prevalence of deformations with the lowest laying performance of 140 eggs. Here, an early start of lay and laying performance could possibly be decisive. In commercial lines, the prevalence of deformation in laying hens varied in general between 32% to 50% [3, 14]. Every 10^th^ mature hen investigated here showed severe deformations. Kittelsen et al. [29] found a high prevalence of deformations in the red jungle fowl with 83% (10 out of 12 animals affected). However, to compare these findings, egg size should be set in relation to the animals’ body weight and still, there might be other factors such as a very low laying performance in the red junglefowl (*Gallus gallus*) contributing to differential prevalence. A similar observation might contribute to this perspective as for Maline hens (late onset of lay, small eggs, low egg performance), where the prevalence for e.g., deviations was low.

Environmental factors might impact the occurrence of KBD. Mature hens of seven breeds and pullets of one breed were kept on two or three farms. For some breeds, the differences in KBD prevalence were large between farms. For example, no KBD was observed in Dresdner hens on farm F5, but 71% were affected on farm F6. Some parameters such as group size and perch material were the same, but perch height was increased 20 cm in farm F6 compared to farm F4. We found comparable differences on farms F5 and F6 for East Frisian Gull, again with farms differing in perch height in the same way, but also with different group sizes applied. For the Marans, the prevalence of KBD was similar between farms but the type of damage was different with more tip damage in farm F7 compared to farm F5 and F6. These results indicate that factors other than breed, such as housing, feeding, or week of age, contribute to the overall occurrence of KBD. Unfortunately, this information was not available for the chickens of our study. In addition, the rearing conditions influence the prevalence of KBD as well, but were unknown for the animals of our study. However, the main reason for our investigation was not the detection of influencing factors, but to find out if they suffer also from KBD.

Our results partly contradict Kittelsen et al. [28], who found low fracture prevalence in four Norwegian local breeds (Icelandic Landrace, NorBird8, Minorka, Roko). The most selected breed, NorBird8, with an early onset of lay, showed the highest prevalence of KBD. The fractures were mainly found at the tip. None of the roosters studied in the Norwegian study showed fractures, whereas we found fractures in every fourth rooster. In contrast to our assessment on chickens kept in free-range systems, all hens in the study of Kittelsen et al. [28] were housed in enriched cages. Rufener and Makagon [36] illustrate that chickens in cages have lower prevalence of fractures than in free-range systems, which could explain the different findings in Norway and Germany, although different breeds were assessed. Here, housing conditions varied also based on the farm resulting in different flock sizes, perch heights, barn structuring, management, etc.. The prevalence of fractures in the red jungle fowl (G*allus gallus*) that Kittelsen et al. [29] report were low (1 hen affected = 8.3%, no rooster affected) and were consistent with those of the pure breeds (*Gallus gallus domesticus*) investigated in a different study [28]. Kittelsen et al. [29] suggested that low egg weight, as it is the case for the red jungle fowl (*Gallus gallus*), might reduce the prevalence of fractures. This assumption is in line with the study by Thøfner et al. [21] who found the early onset of lay influencing the risk of fractures. Our data do neither support nor refuse these findings as we found less KBD in Sulmtaler with a late onset of lay, but not in Bielefelder which start egg laying at a comparable age. Although Thøfner et al. [21] state that heavier eggs result in higher fracture prevalence, whereas Rufener et al. [4] did not find a relationship between egg quality (egg mass, shell breaking strength, shell width) and fractures in commercial strains. Kittelsen et al. [28] conclude from their results that the overall low number of fractures in non-commercial local breeds may involve genetic factors, and thus selective breeding may help reduce susceptibility to fractures.

Our results concerning the severity of damage are in line with Hocking. [38], who compared commercial and local lines for laying performance, egg quality, and bone stability. They found significant genetic variability between commercial lines for bone density and a moderate amount of genetic variability for bone strength. Commercial lines had fragile bones compared to local lines. At the beginning of the selection of today’s high performing layer lines, Warren [39] showed that the tendency to develop deformations was inherited, and that genotype-environment interaction had a great effect. Like in Eusemann et al. [15], we could not generally state that breeds with high laying performance had more KBD, as we only had general information from the literature about egg production of the breeds and not a flock specific assessed laying performance. This, including breed-specific or even individual data on laying performance, would substantially contribute to the discussion on factors driving KBD.

Several studies found different results for the influence of laying performance. On the one hand, Jung et al. [5] and Eusemann et al. [23] found correlations between laying performance and prevalences in the first study for KBD in general, in the second study for fractures. In addition, Eusemann et al. [10] used deslorelin acetate which prevents egg production and found a correlation between suppressed laying performance and decreased incidence of fractures. On the other hand, no correlation between laying performance and keel bone fractures was found in the investigation of Gebhardt-Henrich and Fröhlich [32] and Heerkens et al. [14]. In our study, the breeds Brabançonne and Sulmtaler with a moderate laying performance of expected 180 eggs per year showed no palpable fractures of the keel bone. In contrast to other livestock, there is a clear distinction between show and commercial breeding in chickens. The local breeds are nowadays selected based on their show value leading to a relaxed selection over the last decades and, therefore, decreased production performance. In addition, it can be assumed that the egg production of the investigated breeds is even lower in reality compared to the performance stated in the literature.

Sexes differed in the prevalence of KBD. We found severe deformations of score 2 with more than 1 cm deviation from the straight axis in 20 and tip damage in 82 (out of 289) mature hens. Also, one (out of 18) rooster was affected by tip damage, but none was affected by a severe deformation. Although the prevalence of KBD in roosters was lower, the finding of a tip damage was unexpected as this kind of damage is intensively discussed in the context of an early onset of lay which cannot be the case for rooster. Our study also showed that about one third of the mature hens were presumably affected by fractures along the keel bone detected during palpation. In total, 4 (out of 18) roosters did also show palpable fractures. Although this assumption is based on a very low sample size, roosters seem to be less affected by KBD.

The current study indicates that local breeds might be affected by KBD at a same level as the commercial laying lines, although the egg production of local lines is much lower. Even rooster and pullets showed deformations and palpable fractures. However, the prevalence was lower than in commercial laying hens. In addition, most breeds showed considerable differences in KBD prevalence depending on the farm. Still, individual housing system, management, and also feeding have a high impact on the prevalence of keel bone damage. Not only farmers producing with hens of commercial lines, but also keepers of local breeds should pay attention to the condition of the keel bone and be trained about preventive measures.

To determine differences between and within breeds and trace genetically caused differences, a uniform environment with a higher number of animals would be necessary. Especially the breeds with low prevalence, like Malines and Brabançonne, should be investigated in further research with higher sample sizes and under the same environmental conditions aiming to reveal predispositions for better keel bones and, therefore, better animal welfare.

### Institutional review board statement

The study was carried out in accordance with the German animal protection act (implementing the Directive 2010/63/EU on the protection of animals used for scientific purposes) and the Guidelines for Ethical Treatment of Animals in Applied Animal Behavior and Welfare Research of the International Society for Applied Ethology (2017). The hens were reared and kept for production for human consumption according to national law and guidelines, and not for experimental purposes. They did not undergo any experimental procedure and were only monitored by non-invasive clinical scoring.
